# Supplementary figures and images for: Gut microbiota diversity across ethnicities in the United States
Source: PLoS Biol. 2018 Dec 4;16(12):e2006842. doi: 10.1371/journal.pbio.2006842 (PMC6279019; doi:10.1371/journal.pbio.2006842)

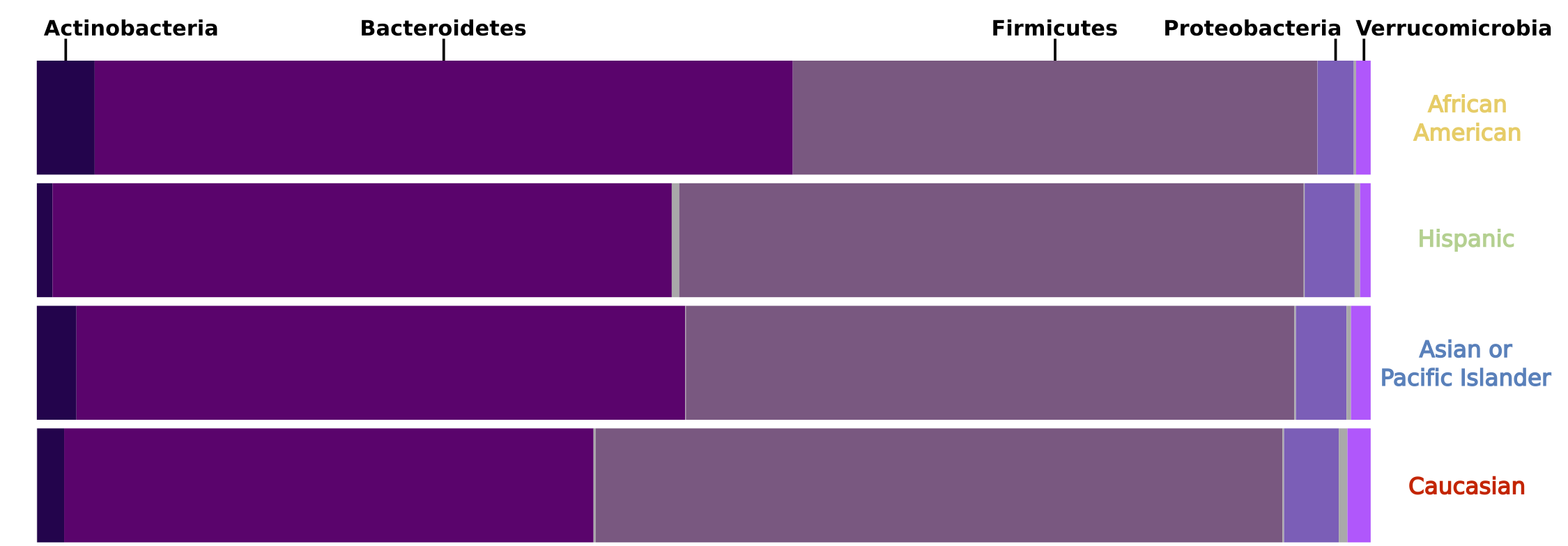

Supplement: S1 Fig — (TIFF) [file pbio.2006842.s001.tiff]

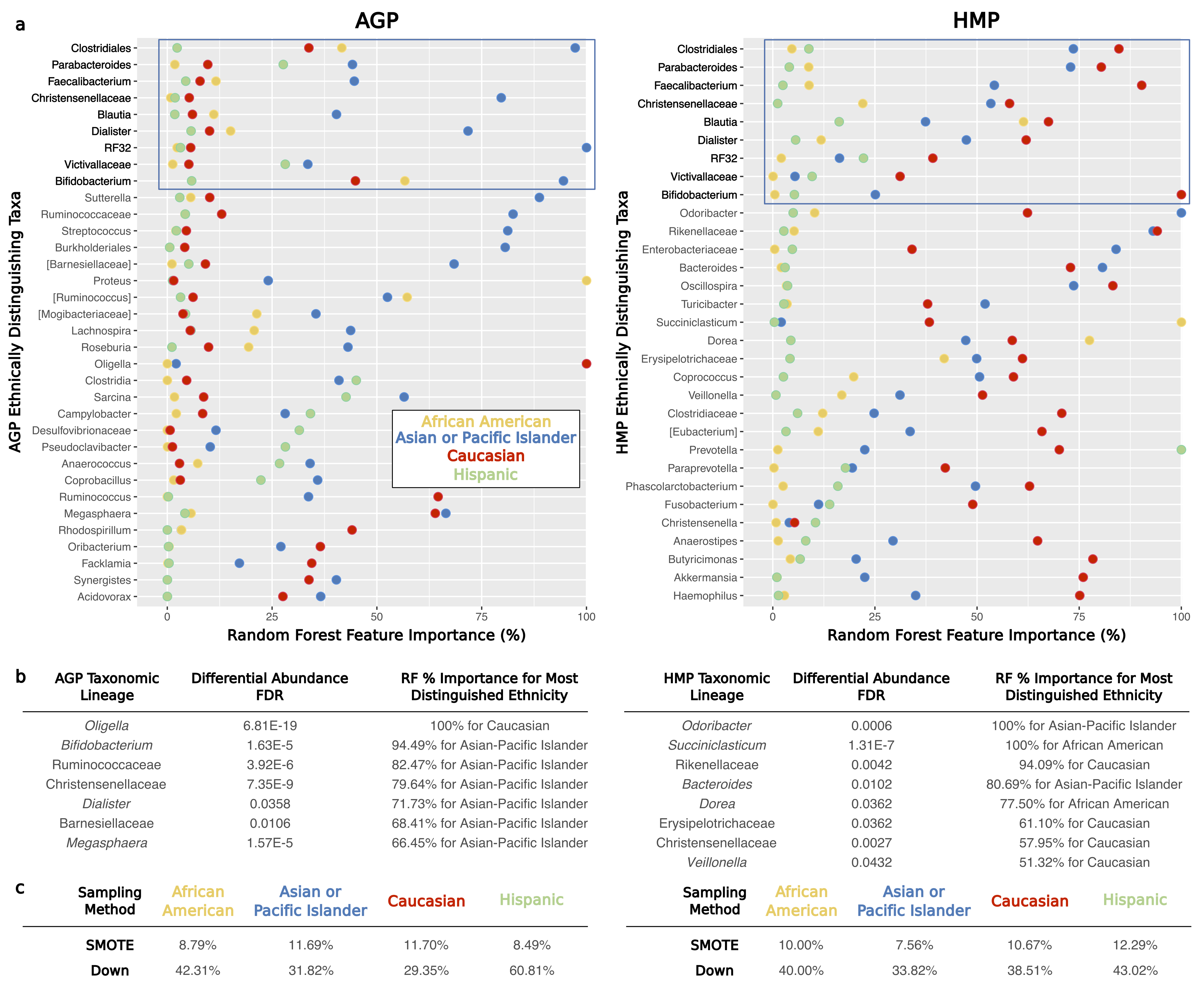

Supplement: S2 Fig — (A) Importance of taxa for predicting each ethnicity using RF models with SMOTE sampling approach are shown as percentage contributions, highlighted by color for each ethnicity. Among the 10 most important taxa for each ethnicity, nine overlap between the AGP and HMP data sets (highlighted by the blue rectangular box); however, which ethnicity they best distinguish varies between the two data sets. (B) Taxa that are distinguishing in RF models and have distinguishing differential abundance in S5 Table. The FDR corrected significance for Kruskal–Wallis tests of differential abundance and the percent importance for the most distinguished ethnicity of each in RF models are shown. (C) Out-of-bag error percentages for the final RF classifier that was built using the optimal model parameters obtained from cross-validation approach corresponding to each ethnicity and sampling procedure for both AGP and HMP data sets. AGP, American Gut Project; FDR, false discovery rate; HMP, Human Microbiome Project; RF, random forest; SMOTE, synthetic minority oversampling technique (TIFF) [file pbio.2006842.s002.tiff]

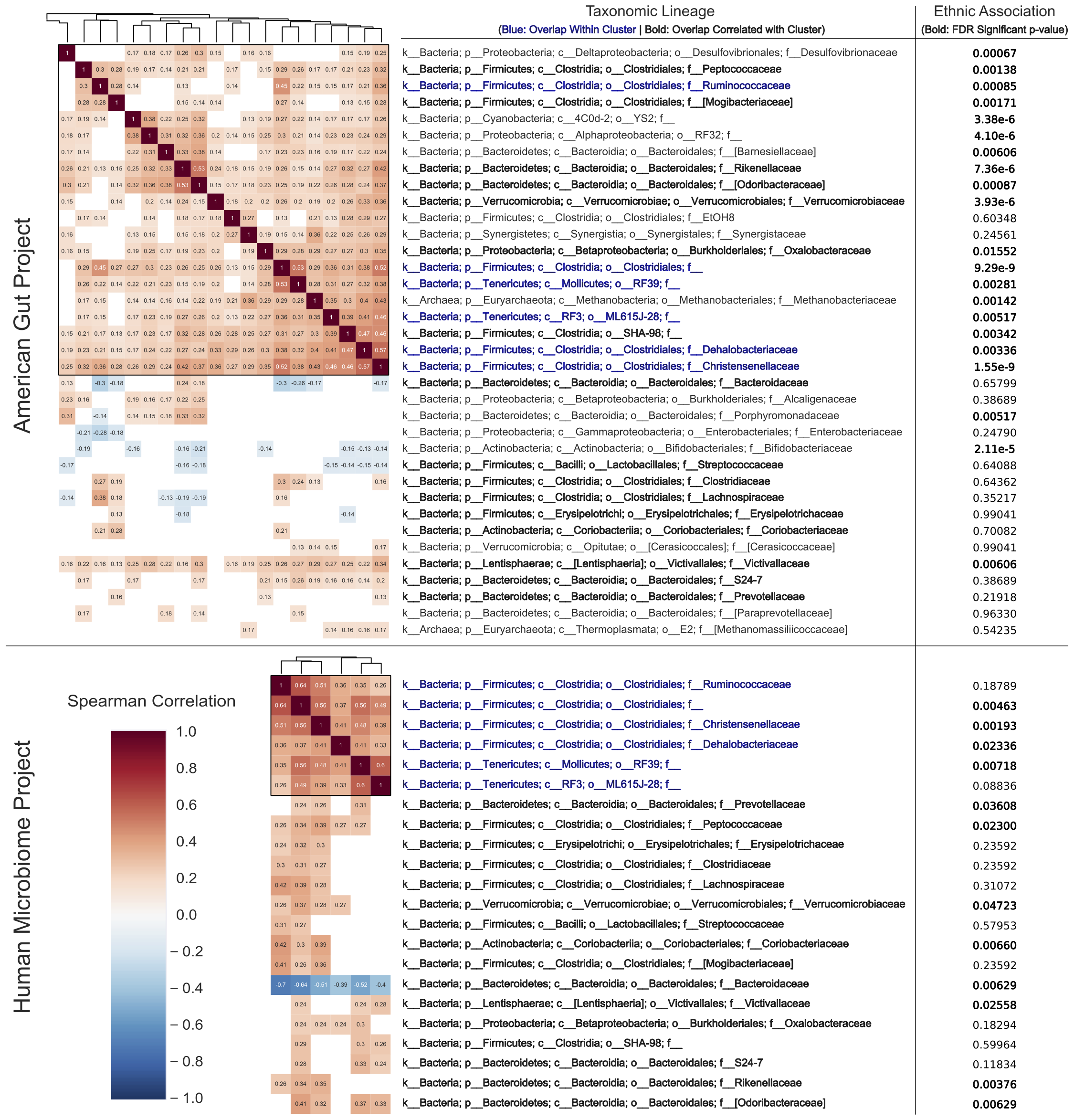

Supplement: S3 Fig — Spearman correlation cluster maps of bacterial abundance for families in the AGP and HMP. Numbers within boxes depict the spearman correlation value with heatmap coloration from blue negative correlation (−1), white no correlation (0), to red positive correlation (1). Positions have been masked based on Bonferroni significance <0.05 for the total cluster map of all microbial families. Taxa within boxes were identified as a highly correlated cluster, and taxa outside the boxes share multiple correlations with those within the cluster. Blue taxonomic names indicate overlap of taxa within boxes of both the AGP and HMP, while black indicate multiple correlations with the clusters in both data sets. The ethnic association column depicts FDR corrected p-values from Kruskal–Wallis tests in S5 Table, which are bolded if <0.05. AGP, American Gut Project; FDR, false discovery rate; HMP, Human Microbiome Project. (TIFF) [file pbio.2006842.s003.tiff]

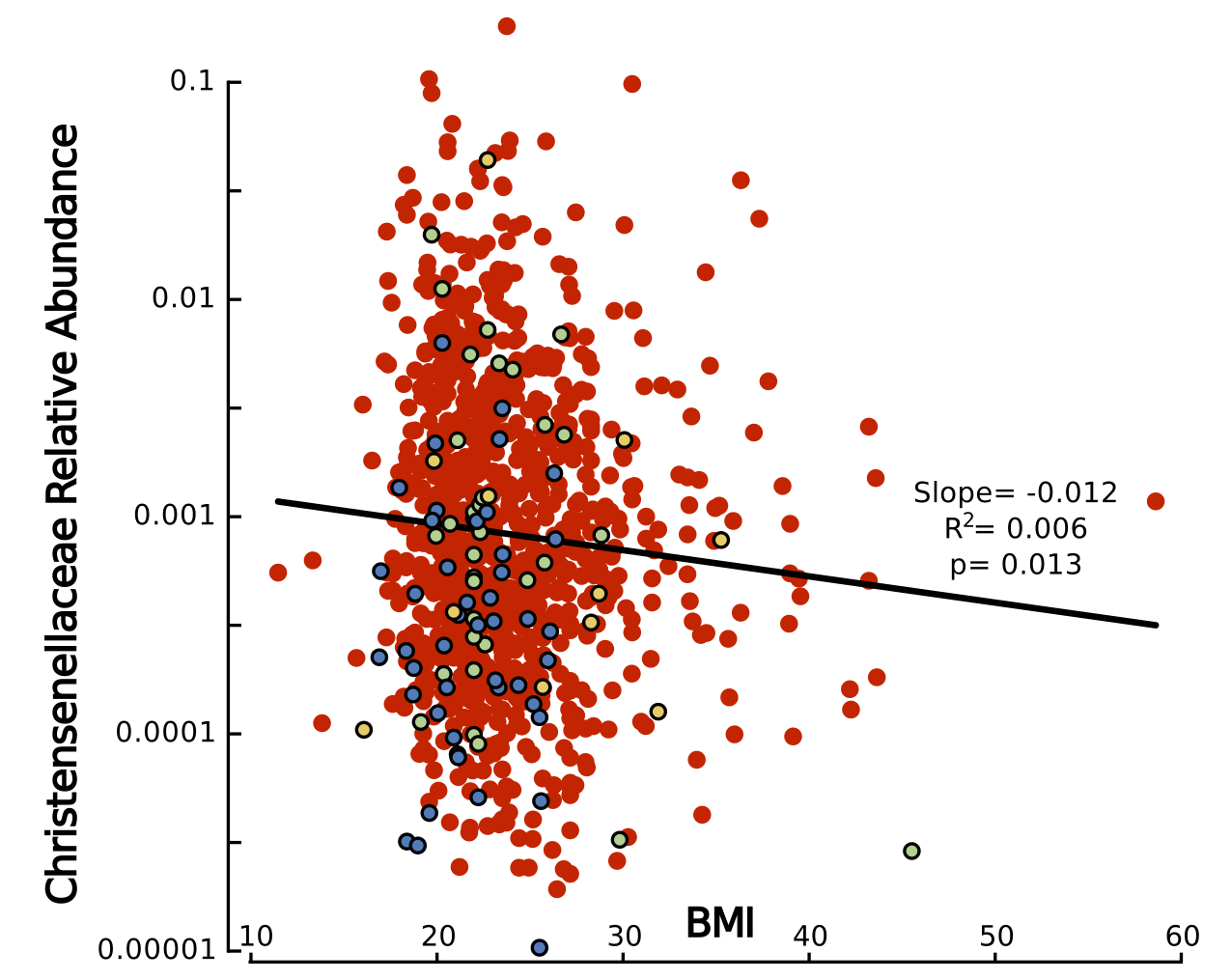

Supplement: S4 Fig — The relationship for each individual between log10 transformed Christensenellaceae abundance on the y-axis and BMI on the x-axis, with statistics slope, R2, and p fit with a linear regression. Coloration of each point indicates ethnicity: yellow, African American; blue, Asian-Pacific Islander; green, Hispanic; red, Caucasian. BMI, body mass index. (TIFF) [file pbio.2006842.s004.tiff]
